# The Influence of Intralayer Porosity and Phase Transition on Corrosion Fatigue of Additively Manufactured 316L Stainless Steel Obtained by Direct Energy Deposition Process

**DOI:** 10.3390/ma15165481

**Published:** 2022-08-09

**Authors:** Maxim Bassis, Tomer Ron, Avi Leon, Abram Kotliar, Rony Kotliar, Amnon Shirizly, Eli Aghion

**Affiliations:** 1 Department of Materials Engineering, Ben-Gurion University of the Negev, Beer-Sheva 8410501, Israel; 2 A. Kotliar Ltd., LWS Laser Welding Solutions, Haifa 32000, Israel

**Keywords:** additive manufacturing, direct energy deposition, wire laser additive manufacturing, 316L steel, fatigue, corrosion

## Abstract

A direct energy deposition (DED) process using wires is considered an additive manufacturing technology that can produce large components at an affordable cost. However, the high deposition rate of the DED process is usually accompanied by poor surface quality and inherent printing defects. These imperfections can have a detrimental effect on fatigue endurance and corrosion fatigue resistance. The aim of this study was to evaluate the critical effect of phase transition and printing defects on the corrosion fatigue behavior of 316L stainless steel produced by a wire laser additive manufacturing (WLAM) process. For comparison, a standard AISI 316L stainless steel with a regular austenitic microstructure was studied as a counterpart alloy. The structural assessment of printing defects was performed using a three-dimensional non-destructive method in the form of X-ray microtomography (CT) analysis. The microstructure was evaluated by optical and scanning electron microscopy, while general electrochemical characteristics and corrosion performance were assessed by cyclic potentiodynamic polarization (CCP) analysis and immersion tests. The fatigue endurance in air and in a simulated corrosive environment was examined using a rotating fatigue setup. The obtained results clearly demonstrate the inferior corrosion fatigue endurance of the 316L alloy produced by the WLAM process compared to its AISI counterpart alloy. This was mainly related to the drawbacks of WLAM alloys in terms of having a duplex microstructure (austenitic matrix and secondary delta-ferrite phase), reduced passivity, and a significantly increased amount of intralayer porosity that acts as a stress intensifier of fatigue cracking.

## 1. Introduction

Additive manufacturing (AM) technologies are primarily developed to rapidly produce complex geometries without the need for additional machining and post-processing procedures [1,2]. This has been commonly achieved using powder bed fusion (PBF) technologies such as selective laser melting (SLM) and electron beam melting (EBM) [3,4,5,6,7,8,9,10,11,12,13,14]. However, the main limitation of PBF technologies relates to their inability to produce large parts at an affordable cost [15,16]. To address this limitation, a branch of AM technologies has been developed in the form of the direct energy deposition process (DED) [17,18,19,20,21]. This process can use wire or powder as the feedstock material to produce large components with a significantly increased deposition rate of up to 3 kg/h compared to only about 0.1 kg/h in the case of common powder bed fusion (PBF) processes [22,23,24]. One of the attractive methods of DED is the wire laser additive manufacturing (WLAM) process. This method uses welding wire as a feedstock material and a high-powered laser, which is controlled by a computerized numerical control (CNC) system. The principle of this method is to transfer the feedstock material through a jet nozzle onto a metal substrate and to melt it by a laser beam. Consequently, the molten layers fuse one on top of the other (layer-by-layer), and the process continues until the part is fully constructed [25,26]. This method can be used with a large variety of alloys and without any limitation relating to the size of the printing chamber.

Currently, most of the research activities using the WLAM process are related to Ti-based alloys, nickel-based superalloys, and carbon steels [14,27,28], while relatively few studies have been dedicated to austenitic stainless steels [11]. The attractive mechanical properties and corrosion resistance of austenitic stainless steel, and in particular of the 316L alloy, make them a promising choice as a structural material for AM technologies in common applications relating to the medical, nuclear, and aerospace industries [29]. An important aspect of the 316L alloy that is critical with regard to AM production is the safety and adequacy of the printed component in terms of fatigue endurance. Most of the research studies related to this issue examined the fatigue performance in an air atmosphere. Solberg et al. [30] evaluated the effect of internal porosity and surface roughness on the fatigue endurance of a 316L alloy produced by an SLM process. According to their study, the high cycle fatigue (HCF) failure at a low load level was due to surface defects, while at higher load levels, the failure occurred due to internal defects, mainly in the form of complex porosity. Dass and Moridi [31] considered porosity obtained in AM-DED processes as a critical printing defect that can have a significant effect on the mechanical properties. They distinguished between interlayer porosity and intralayer porosity. While interlayer porosity is related to insufficient heating, which creates an un-melted region and is also known as lack of fusion [6], intralayer porosity is connected to the use of an inert shielding gas that promotes gas entrapment. Usually, intralayer porosity will have a spherical shape and tends to evolve at arbitrary sites with a relatively reduced solidification rate. Shrestha et al. [32] tested the effect of layer orientation and surface roughness on the fatigue behavior of the 316L alloy produced via laser beam PBF. They found that the detrimental effect on fatigue resistance was mainly due to the presence of large internal defects and building orientation. Blinn et al. [33] examined the fatigue behavior of a 316L alloy produced by a laser-DED process using powder and wire as the feedstock materials. Their results showed that, in both cases, crack initiation took place at nonmetallic inclusions that were induced by the AM process. In addition, they indicated that the differences in the mechanical properties of the tested alloys that affect the fatigue behavior were related to the different content of delta-ferrite in the microstructure.

Altogether, although the fatigue behavior of AM 316L stainless steel in air is fairly well understood, the knowledge relating to the combined effect of fatigue conditions and a corrosive environment is extremely limited. Hence, the aim of this study is to evaluate the effect of printing defects and phase transition on the corrosion fatigue of 316L stainless steel produced by the AM-DED process using wire as the feedstock material.

## 2. Experimental Procedure

### 2.1. Preparation of the Tested Specimens

The raw material in the form of rods (110 mm length and 12 mm radius) for the tested specimens was produced by a custom-built WLAM setup using 316L stainless steel wire (1.2 mm diameter) and pure nitrogen (99%) as the protective gas atmosphere. The configuration of the rods was programmed using a CAD model. The laser power was 1.5 kW; the wire feeding speed was 12 mm/sec; the traveling speed of the welding head was 7 mm/sec. These production parameters were able to produce a deposition rate of about 200 gr/h and generate a layer thickness of 0.7 mm. The printing stage was cooled by distilled water throughout the production process. For comparison, a counterpart alloy made from standard AISI 316L stainless steel with an austenitic microstructure was examined.

### 2.2. Microstructure Evaluation

Microstructure assessment of the tested specimens was conducted by optical and scanning electron microscopy (SEM-JEOL 5600, JEOL Ltd., Tokyo, Japan). The metallographic analysis incorporated grinding and polishing up to 0.25 µm using a colloidal silica suspension and two-stage etching in (i) 5 mL HNO_3_ (70%) and 9 mL HCL (32%) and (ii) 5 mL ethanol for about 30 s. This was followed by an additional immersion stage in 5% Nital solution to disclose the presence of a ferritic phase.

### 2.3. X-ray Microtomography (CT) Analysis

The CT analysis aimed at evaluating structural printing defects that can affect the fatigue endurance of WLAM 316L stainless steel. This 3D non-destructive method was carried out using a micro CT (EasyTom XL, RXSolutions, Chavanod, France) equipped with a Mechanic ULTRA 230 KV, 200 W open tube to obtain a 5 μm resolution.

### 2.4. Fatigue Testing

**Figure 1:**
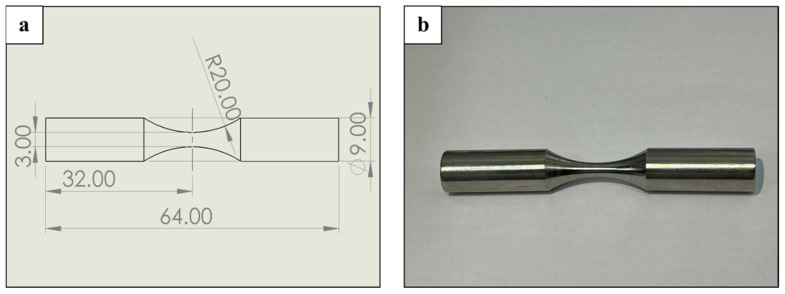
Description of the fatigue specimen. (**a**) Dimensions of the specimen. (**b**) Photo of the specimen.

The fatigue endurance of WLAM 316L stainless steel and a counterpart AISI 316L alloy was examined in terms of an S-N curve using a SM1019 rotating fatigue machine (TecQuipment Ltd., Nottingham, U.K.). The specimens’ length was 64 mm, and the neck thickness was 3 mm, as shown in Figure 1. The fatigue specimens were tested under a simulated corrosive environment in the form of a 3.5% NaCl solution. During testing, this solution mainly covered the neck section of the fatigue specimen by continuous dropping of fresh solution. Furthermore, to evaluate the effect of the corrosive environment on the fatigue endurance, parallel tests were carried out in an air atmosphere.

### 2.5. Electrochemical Characteristics and Corrosion Performance

The electrochemical characteristics and corrosion performance of the tested alloys were evaluated by cyclic potentiodynamic polarization (CPP) analysis and immersion tests. The CPP tests were conducted using a Bio-Logic SP-200 potentiostat (Bio-Logic Science Instruments, Grenoble, France) with EC-Lab system software and a standard three-electrode cell. The scanning rate of the CPP analysis was 0.5 mV/s, while using saturated calomel as a reference electrode and 3.5% NaCl as a simulated corrosive solution. The corrosion solution for the immersion test was 1 M of HCl. This solution was selected to accelerate corrosion degradation and magnify the differences in corrosion behavior between WLAM 316L stainless steel and its counterpart AISI 316L alloy [34,35]. Both CCT and immersion tests were carried out at ambient temperature, and the localized corrosion attack in terms of pitting corrosion characteristics was evaluated using the ASTM G46 standard.

## 3. Results

**Figure 2:**
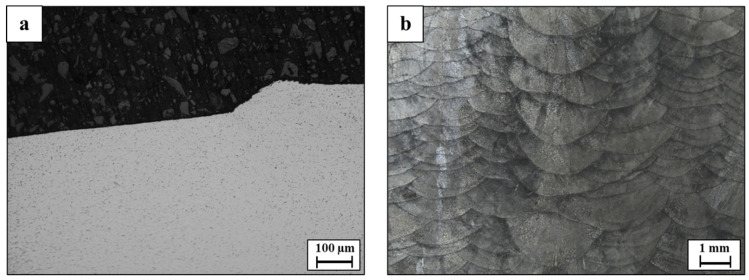
General appearance of WLAM 316L stainless steel. (**a**) Typical surface roughness. (**b**) Macrostructure on XZ plane.

The general appearance of WLAM 316L stainless steel samples is shown in Figure 2. This reveals a relatively high surface roughness that was generated due to the rapid deposition rate obtained by the WLAM process [18]. A close up view on the XZ plane shows the distinct tracks of the AM process that tend to have a “fish-scale” morphology due to the combined actions of recoil pressure [36], surface tension, and melt pool shape [37].

**Figure 3:**
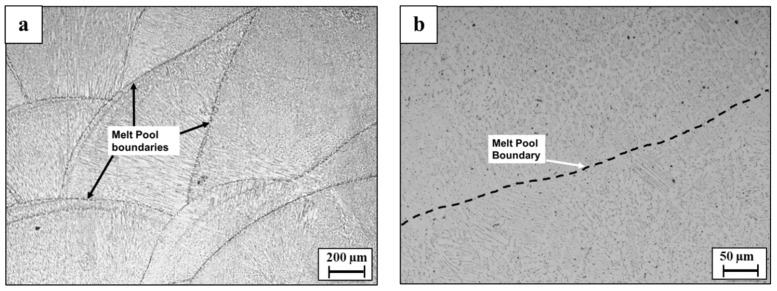
Microstructure of WLAM 316L alloy on XZ plane (**a**,**b**) showing melt pool boundaries at low and high magnifications, respectively.

**Figure 4:**
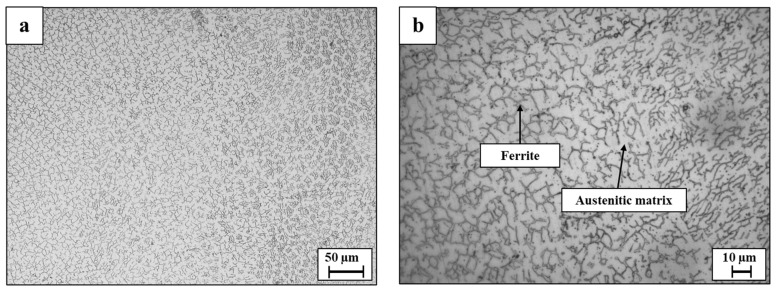
Microstructure of WLAM 316L samples on the XY plane (**a**,**b**) at reduced and high magnifications, respectively, showing austenitic matrix and secondary ferritic phase.

**Figure 5:**
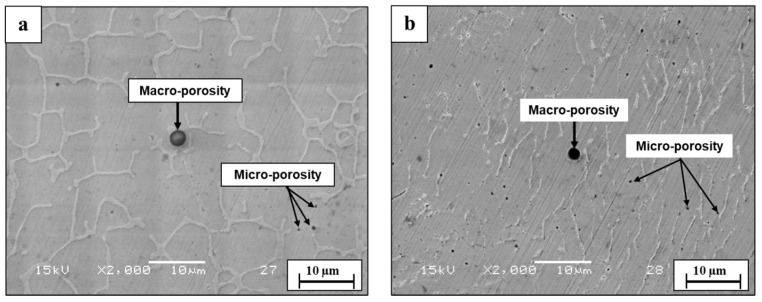
Printing defects in WLAM 316L samples in the form of microporosity and macroporosity. (**a**) XY plane; (**b**) XZ plane.

Microstructure analysis of the WLAM 316L alloy on the XZ plane (printing direction-front cross-section view) clearly showed the presence of melt pool boundaries, along with an epitaxial solidification tendency of a secondary phase [38,39], as introduced in Figure 3. Further analysis on the XY plane (printing path-top cross-section view) revealed the existence of a duplex microstructure composed of an austenitic matrix and a secondary ferritic phase [13], as expected from the Schaeffler diagram [40] and introduced in Figure 4. This microstructure of WLAM 316L alloy is in contrast to the pure austenitic morphology of its counterpart AISI 316L alloy, which has the same chemical composition [6,40]. In terms of printing defects, Figure 5 clearly demonstrates the presence of macroporosity and microporosity with sizes of 1 µm and up to 6 µm, respectively, in both the XY and XZ planes. This porosity can be identified as “keyholing porosity” or intralayer porosity, usually generated due to the use of an inert protective gas atmosphere and consequent gas entrapment [32], as can be expected from DED processes [41,42,43]. It should be pointed out that such porosity can have a significant detrimental effect on the mechanical properties and, in particular, on the fatigue endurance due to the fact that pores can act as fatigue initiation sites.

**Figure 6:**
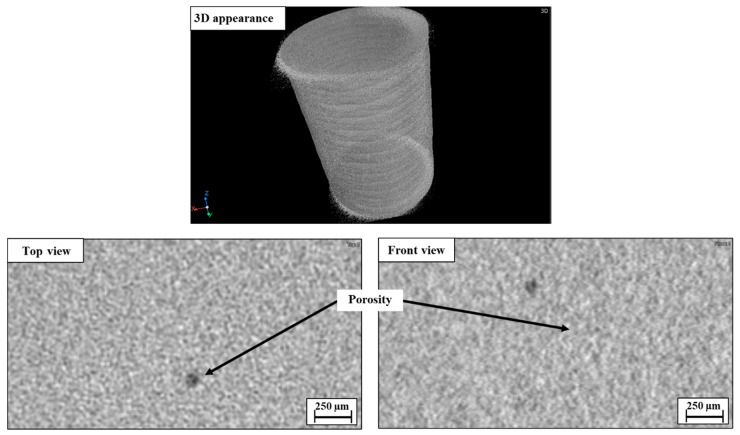
CT analysis of WLAM 316L alloy.

**Figure 7:**
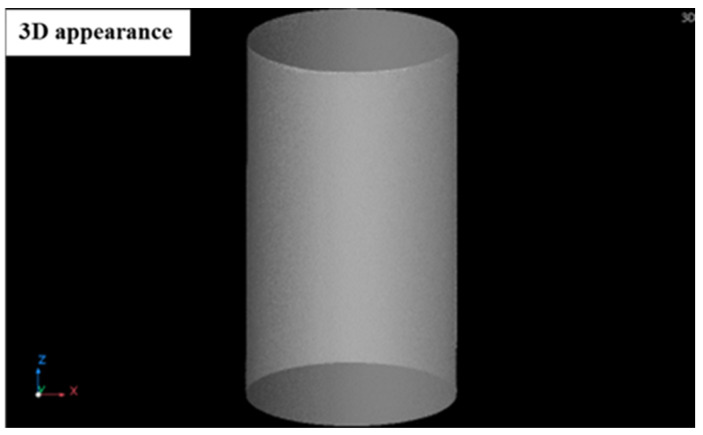
CT analysis of counterpart AISI 316L alloy.

X-ray microtomography (CT) analysis of WLAM 316L and its counterpart AISI 316L alloy is shown in Figure 6 and Figure 7, respectively. This reveals the rough surface of the WLAM alloy compared to the smooth surface of the counterpart alloy that did not include any abnormal defects. Close examination of the WLAM alloy showed the presence of porosity with a spherical shape that is in line with the observation of the electron microscopy analysis shown in Figure 5.

**Figure 8:**
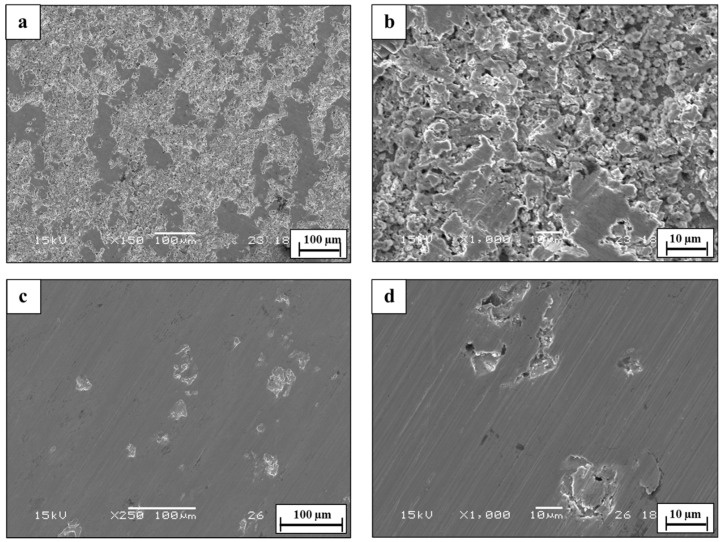
General view of pitting corrosion in tested alloys after immersion test in 1 M HCl solution for 100 h. (**a**,**b**) WLAM 316L alloy; (**c**,**d**) AISI 316L alloy.

The corrosion performance of WLAM 316L and its counterpart AISI 316L alloy in terms of pitting density and the pitting factor after the immersion test in 1 M of HCl solution for 100 h is shown in Figure 8, along with quantitative analysis in Table 1. This reveals that both alloys suffered from a localized corrosion attack, mainly in the form of pitting. However, the extent of the corrosion attack in the WLAM 316L alloy was significantly increased compared to its counterpart AISI 316L. For example, the pitting density in WLAM 316L was 14-times higher than that obtained by the counterpart AISI 316L alloy. This could be mainly related to the inherent process defects of the WLAM 316L alloy and the fact that this alloy contains a ferritic phase with reduced corrosion resistance [6,11].

**Figure 9:**
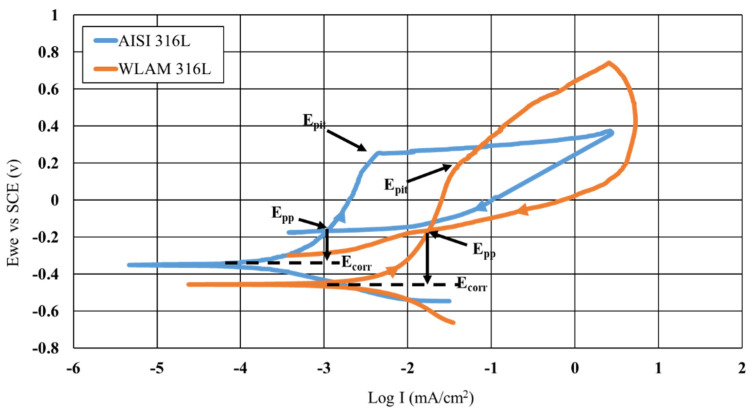
Cyclic potentiodynamic polarization analysis of WLAM 316L stainless steel and its counterpart AISI 316L alloy.

CPP analysis basically supports the results obtained by the immersion test. Typical cyclic anodic polarization curves are shown in Figure 9, along with the derived electrochemical parameters that are introduced in Table 2. Both alloys exhibited polarization curves with active–passive transition, as expected from 316L stainless steel [44,45]. However, the polarization curve of the WLAM 316L alloy was relatively displaced to higher current densities, which is an indication of reduced corrosion resistance. This was supported by the corrosion rate measurements using Tafel extrapolation, which indicated that the corrosion rate of the WLAM 316L alloy was nearly two orders of magnitude higher compared to its counterpart alloy. In addition, the E_pit_ of WLAM 316L alloy, which represents the potential where the current density rapidly intensifies to form pits, was relatively reduced, which is an indication of decreased passivity in terms of the external film stability [46]. This was also supported by the relatively higher protection potential (E_pp_) of the AISI 316L alloy and the increased difference of E_pit_-E_corr_ (0.6 V vs. 0.58 V) that reflect higher resistance to pitting corrosion [47]. The relatively reduced corrosion resistance of the WLAM 316L alloy can be related to printing defects such as porosity and a lack of fusion, which inherently damage the stability of the oxide layer [46,47].

**Figure 10:**
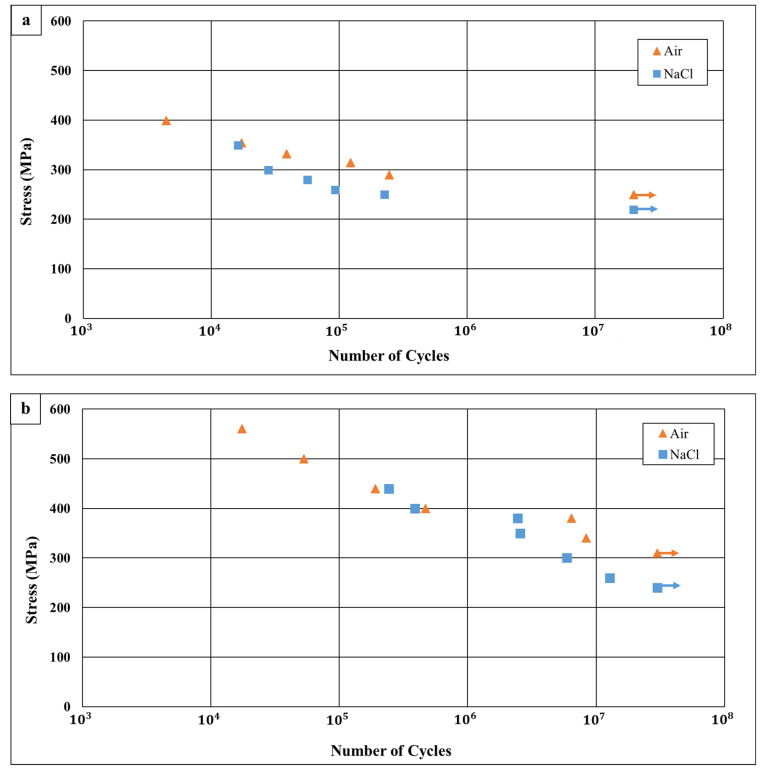
S-N curves obtained in air and in 3.5% NaCl solution. (**a**) WLAM 316L alloy. (**b**) Counterpart AISI 316L alloy.

**Figure 11:**
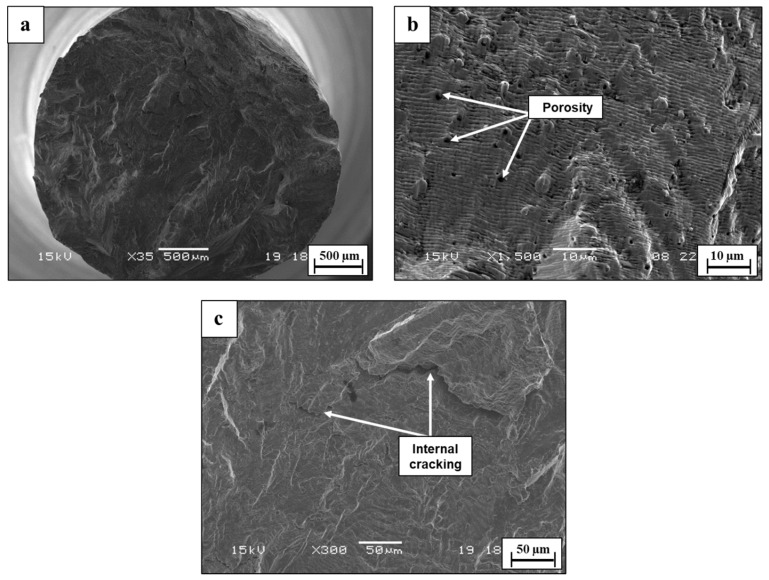
Fractography analysis of WLAM 316L alloy in air (S = 290 MPa, N = 245,700). (**a**) General view (**b**,**c**) close-up view.

**Figure 12:**
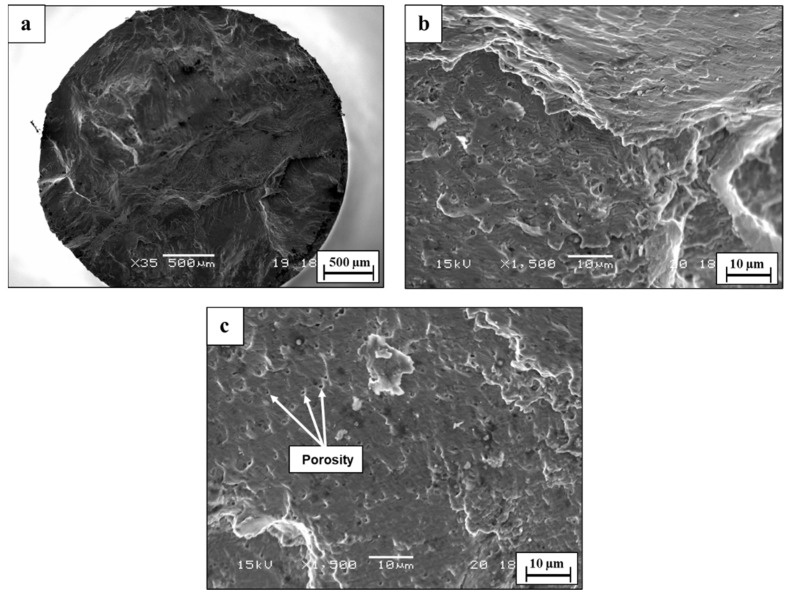
Fractography analysis of WLAM 316L alloy in 3.5% NaCl solution (S = 250 MPa, N = 225,722) (**a**) General view (**b**,**c**) close-up view.

**Figure 13:**
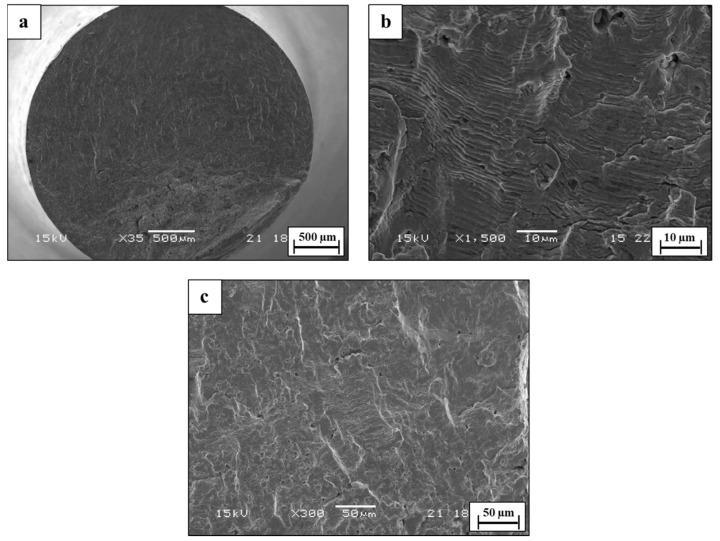
Fractography analysis of counterpart AISI 316L alloy in air (S = 340 MPa, N = 8,398,345). (**a**) General view (**b**,**c**) close-up view.

**Figure 14:**
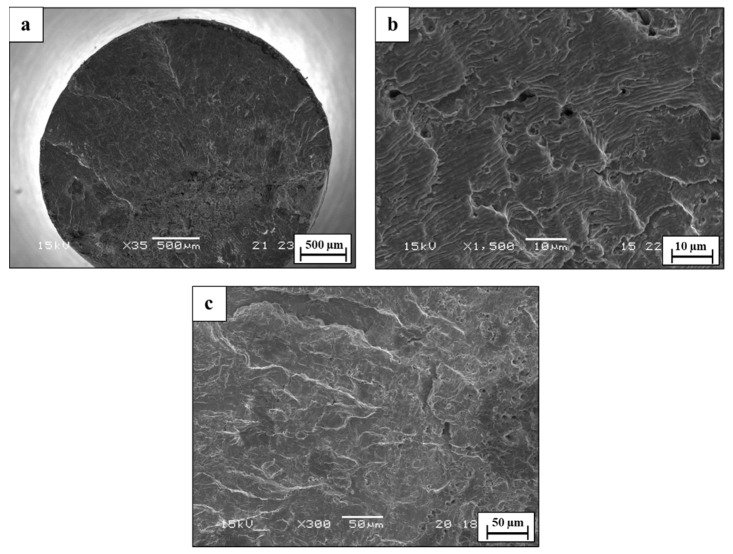
Fractography analysis of counterpart AISI 316L alloy in 3.5% NaCl solution (S = 300 MPa, N = 5,900,000). (**a**) General view (**b**,**c**) close-up view.

The fatigue characteristics of WLAM 316L and its counterpart AISI 316L alloy, examined in terms of S-N curves in air and in 3.5% NaCl solution, are shown in Figure 10, along with selected fractography analysis presented in Figure 11, Figure 12, Figure 13 and Figure 14. This reveals that the endurance limit of WLAM 316L was significantly reduced compared to its counterpart AISI 316L alloy in both air (250 MPa vs. 305 MPa, respectively) and 3.5% NaCl solution (215 MPa vs. 250 MPa, respectively). Assuming that a rough estimation of the fatigue limit is at 2 × 10^6^ cycles [48,49], the obtained S-N results related to the WLAM 316L alloy in air were in good agreement with the results of Blinn et al. [33]. Relating to the detrimental effect of the corrosive environment on fatigue endurance, it was evident that both alloys suffered from quite a similar reduction of about 15% compared to tests carried out in air.

The fractography analysis of the WLAM 316L alloy in air demonstrated a relatively brittle fracture with a complex appearance that is related to the inherent macrostructure of the alloy, as shown in Figure 11. In this case, the melt pools practically form a discontinuous structure within the bulk material, which, under fatigue conditions (alternative loading), causes striations to shift from a coarse to fine configuration. This phenomenon was intensified under the presence of porosity and other printing defects, which can stimulate internal cracking and shift the velocity of crack propagation. The reduction in fatigue endurance can also be related to the decreased load bearing at the cross-section due to printing defects, especially porosity and a lack of fusion [50]. The fractography analysis in 3.5% NaCl solution magnified the complex fracture nature of this alloy, as shown in Figure 12. This was mainly related to an increased number of crack-initiating sites generated by the pitting corrosion attack at the external surface. In addition, striations were hard to detect due to the etching effect of the corrosive solution. Altogether, the interactive combination of more crack initiation sites and internal cracking due to the presence of printing defects created a fracture surface with a multi-cracking appearance, which consequently reduced the fatigue endurance.

In comparison, the fatigue fracture of the counterpart AISI 316L alloy in air demonstrated a relatively ductile failure with a typical “Fish-eye” structure [51,52], as shown in Figure 13. The crack-initiating site at the north pole was followed by a large smooth area with visible striations that represent the crack propagation pathway, while the final overload failure was clearly located at the south pole. The fractography analysis in 3.5% NaCl solution indicated that the mode of failure remained mostly ductile, although the final overload rupture at the south pole was relatively enlarged (Figure 14).

## 4. Discussion

According to this study, the inherent differences between WLAM 316L and its counterpart AISI 316L alloy that affect their corrosion fatigue endurance can be related to (i) major differences in microstructure and phase composition, (ii) structural defects in the form of intralayer porosity, and (iii) electrochemical behavior. While the microstructure of the counterpart alloy is purely austenitic, the hybrid structure of the WLAM alloy was composed of an austenitic matrix and a secondary ferritic phase. According to Gou et al. [40], the creation of the ferritic phase in the WLAM alloy can be attributed to the solidification characteristics of the melt pools. This relates to the assumption that, during solidification of the melt pool, there is heat dissipation from the melt pool to the previously solidified layer. Subsequently, the cooling rate at the external edge of the melt pool is relatively reduced and the Cr/Ni ratio is increased. Hence, according to the Schaeffler diagram [53], the local reduction in Ni that stabilizes the austenitic structure triggers the formation of the secondary ferritic phase. As for the presence of structural defects, and in contrast to the counterpart alloy, the WLAM alloy contained a significantly increased amount of intralayer porosity in the form microporosity and macroporosity with a spherical shape, as found in the microscopy observation (Figure 5) and CT analysis (Figure 6). According to Dass et al. [31], this intralayer porosity is mainly related to the usage of the inert protective gas that stimulates gas entrapment. Intralayer porosity occurs at arbitrary locations and tends to take place at regions with reduced solidification cooling rates. With regard to the electrochemical behavior and Tafel extrapolation, it was evident that the corrosion rate of the WLAM 316L alloy was significantly increased compared to its counterpart alloy. This was also supported by the immersion test and can mainly be attributed to the presence of internal porosity and to partial phase transition from austenite to ferrite, which has a relatively reduced corrosion resistance [54]. Consequently, the combined effect of internal porosity and the generation of a ferritic phase promotes localized corrosion attack, mainly in the form of pitting.

The influence of the above inherent differences between WLAM 316L and its counterpart alloy had a substantial effect on their fatigue endurance in both air and a corrosive environment. This was clear from the results of the S-N curves and fractography analysis (Figure 10, Figure 11 and Figure 12), which demonstrated that the fatigue endurance of the WLAM 316L alloy was comparatively reduced. This observation was in accord with the results obtained by Blinn et al. [33] in air. To explain the relatively reduced fatigue endurance of WLAM 316L, one should understand the solidification mechanism of the melt pools that are the cornerstone for the AM-DED layer-by-layer construction. According to Guo et al. [40], stainless steel with a fully austenitic microstructure can be formed at a higher cooling rate and low Creq/Nieq ratio, while a duplex microstructure (austenite + ferrite) is obtained at a low cooling rate and high Creq/Nieq ratio. In general, the cooling rate of the melt pool in AM processes with laser beams, as described by Ma et al. [55], is illustrated by the following equation:

$$\frac{\Delta T}{\Delta t}=C\times E\times V^{\frac{1}{2}}\times \delta^{-\frac{3}{2}}$$

where ΔT/Δt is the melt pool cooling rate, C is a constant that relates to the feedstock material, E is the density of the laser power, V is the laser scanning speed, and δ represents the layer thickness. Combining the findings of Guo et al. [40], Schaeffler [53] and Ma et al. [55] can explain the formation of the secondary ferritic phase in the WLAM 316L alloy. In general, it is assumed that the formation of the ferritic phase in the WLAM alloy can be limited if the cooling rate is increased and the Creq/Nieq ratio is reduced. This may be obtained by modifying the printing parameters and by increasing the content of Ni in the feeding wire.

To the best of our understanding, it is believed that the intralayer porosity played a dominant role in reducing the fatigue endurance of WLAM 316L compared to its counterpart alloy in air. This assumption was strongly supported by the relatively brittle fatigue fracture of WLAM 316L, which demonstrated the intensifying effect of intralayer porosity on internal cracking, along with reduced load bearing. This insight is in line with the results of Solberg et al. [30], Wang et al. [56], and Sheridan et al. [57], who found a clear link between the reduced fatigue endurance of and the presence of porosity in stainless steel and superalloys produces by an AM process. In corrosion fatigue conditions, the comparatively reduced fatigue endurance of WLAM 316L can be mainly related to the relatively increased pitting corrosion attack that enhanced crack initiation processes [58]. The contribution of intralayer porosity in terms of enhancing pitting corrosion was also amplified by the fact that the WLAM 316L alloy contains a ferritic phase that has reduced corrosion resistance compared to an austenitic matrix [4,9].

## 5. Conclusions

The fatigue properties in terms of S-N curves showed a significant reduction in corrosion fatigue endurance of the WLAM alloy in comparison to its counterpart AISI 316L. The reduced corrosion fatigue resistance of the WLAM alloy was attributed to the inherent microstructural drawback of the WLAM alloy. This was mainly related to printing defects in the form of intralayer porosity and the duplex microstructure, which was composed of an austenitic matrix and a secondary delta ferrite phase. The modifications in the microstructure of the WLAM alloy were explained in relation to the solidification mechanism of the melt pools that govern the AM-DED layer-by-layer construction. This led to the understanding that the deteriorating influence of the corrosive environment on the fatigue endurance of the WLAM alloy was linked to the presence of intralayer porosity, which promotes pitting corrosion and, consequently, increases the number of crack initiation sites. The pitting corrosion attack was also amplified by the presence of a ferritic phase with relatively reduced corrosion resistance compared to a pure austenitic structure.

## Figures and Tables

**Table 1:** The pitting density and pitting factor of WLAM 316L and its counterpart AISI 316L alloy in 1 M HCl solution.

Material	WLAM 316L	AISI 316L
$\mathrm{Pitting}\;\mathrm{density}\;(\mathrm{pits}/{\mathrm{cm}}^{2}$)	3500	250
Pitting factor	9.28	8.88

**Table 2:** Electrochemical parameters derived from potentiodynamic polarization analysis shown in Figure 9.

Material	Ecorr (V)	Icorr (µA)	C.R (Mmpy)	Epit-Ecorr (V)
AISI 316L	−3.00	0.43	0.004	0.6
WLAM 316L	−4.45	10.27	0.11	0.58

## Data Availability

Experimental data from this study are available from the corresponding author upon reasonable request.
